# High-Throughput Wastewater SARS-CoV-2 Detection Enables Forecasting of Community Infection Dynamics in San Diego County

**DOI:** 10.1128/mSystems.00045-21

**Published:** 2021-03-02

**Authors:** Smruthi Karthikeyan, Nancy Ronquillo, Pedro Belda-Ferre, Destiny Alvarado, Tara Javidi, Christopher A. Longhurst, Rob Knight

**Affiliations:** a Department of Pediatrics, University of California, San Diego, La Jolla, California, USA; b Department of Electrical and Computer Engineering, University of California, San Diego, La Jolla, California, USA; c Department of Biomedical Informatics, University of California, San Diego, La Jolla, California, USA; d Department of Bioengineering, University of California, San Diego, La Jolla, California, USA; e Department of Computer Science & Engineering, University of California, San Diego, La Jolla, California, USA; f Center for Microbiome Innovation, University of California, San Diego, La Jolla, California, USA; Princeton University

**Keywords:** COVID-19, SARS-CoV-2, epidemiology, wastewater

## Abstract

Large-scale wastewater surveillance has the ability to greatly augment the tracking of infection dynamics especially in communities where the prevalence rates far exceed the testing capacity. However, current methods for viral detection in wastewater are severely lacking in terms of scaling up for high throughput. In the present study, we employed an automated magnetic-bead-based concentration approach for viral detection in sewage that can effectively be scaled up for processing 24 samples in a single 40-min run. The method compared favorably to conventionally used methods for viral wastewater concentrations with higher recovery efficiencies from input sample volumes as low as 10 ml and can enable the processing of over 100 wastewater samples in a day. The sensitivity of the high-throughput protocol was shown to detect 1 asymptomatic individual in a building of 415 residents. Using the high-throughput pipeline, samples from the influent stream of the primary wastewater treatment plant of San Diego County (serving 2.3 million residents) were processed for a period of 13 weeks. Wastewater estimates of severe acute respiratory syndrome coronavirus 2 (SARS-CoV-2) viral genome copies in raw untreated wastewater correlated strongly with clinically reported cases by the county, and when used alongside past reported case numbers and temporal information in an autoregressive integrated moving average (ARIMA) model enabled prediction of new reported cases up to 3 weeks in advance. Taken together, the results show that the high-throughput surveillance could greatly ameliorate comprehensive community prevalence assessments by providing robust, rapid estimates.

**IMPORTANCE** Wastewater monitoring has a lot of potential for revealing coronavirus disease 2019 (COVID-19) outbreaks before they happen because the virus is found in the wastewater before people have clinical symptoms. However, application of wastewater-based surveillance has been limited by long processing times specifically at the concentration step. Here we introduce a much faster method of processing the samples and show its robustness by demonstrating direct comparisons with existing methods and showing that we can predict cases in San Diego by a week with excellent accuracy, and 3 weeks with fair accuracy, using city sewage. The automated viral concentration method will greatly alleviate the major bottleneck in wastewater processing by reducing the turnaround time during epidemics.

## OBSERVATION

Wastewater-based epidemiology (WBE) can facilitate detailed mapping of the extent and spread of severe acute respiratory syndrome coronavirus 2 (SARS-CoV-2) in a community and has seen a rapid rise in recent months owing to its cost effectiveness as well as its ability to foreshadow trends ahead of diagnostic testing (1–4). With over 46 million cases reported globally, 9.3 million of which are from the United States, there is an imminent need for rapid, community-level surveillance in order to identify potential outbreak clusters ahead of diagnostic data. Previous studies have reported high levels of correlation between viral concentration in sewage to clinically reported cases in a community with trends appearing 2 to 8 days ahead in wastewater (2, 5). A major bottleneck in large-scale wastewater surveillance is the lack of robust, high-throughput viral concentration methodology. Conventional techniques for viral concentration from wastewater typically employ laborious or time-consuming processes, namely, polyethylene glycol (PEG)-based precipitation, direct filtration, or ultrafiltration methods that severely limit throughput (6). In the present study, we employed an affinity-capture magnetic hydrogel particle (Nanotrap)-based viral concentration method which was incorporated on the KingFisher Flex liquid-handling robot platform robots (Thermo Fisher Scientific, USA), using a 24-plex head to process 24 samples at once in a 40-min run. RNA is then extracted on the same KingFisher system for rapid sample processing. Reverse transcription-quantitative PCR (RT-qPCR) targeting N1, N2, and E gene targets was used for detection and quantification of SARS-CoV-2 RNA. Detection of all three genes in a sample and its replicate (at quantification cycle [Cq] values of <39) was considered positive. All steps from concentration to RT-qPCR plating were performed hands-free and conducted by liquid-handling robots to minimize human error. Using the above pipeline, 96 raw sewage samples were processed in a period of 4.5 h (concentration to RT-qPCR detection/quantification), effectively reducing the processing time by at least 20-fold. The sensitivity of detection of SARS-CoV-2 viral RNA in wastewater by the high-throughput pipeline (i.e., to determine whether individual buildings yield sufficient wastewater signal for high-resolution spatial studies) was established by routinely monitoring the SARS-CoV-2 signatures in the wastewater of a large San Diego hospital building housing active coronavirus disease 2019 (COVID-19) patients (7). This site was used as a positive control to test correlations with caseload on a daily basis. Sewage samples were collected daily for a period of 12 weeks during which time the hospital’s caseload (specific to COVID-19 patients) varied between 2 and 26. SARS-CoV-2 viral gene copies correlated with the daily hospital caseload (*r* = 0.75; see Fig. S1 in the supplemental material), suggesting that the wastewater data could at least be used to identify the peaks. SARS-CoV-2 viral RNA was detected in wastewater on all days sampled. Furthermore, the high-throughput protocol has been used as a part of an on-campus wastewater surveillance for the last few months where data from 70 autosamplers covering individual buildings are sampled and analyzed on a daily basis. The method enabled detection of cases as low as 1 asymptomatic individual in buildings with over 400 occupants (unpublished data [data available on request]).

10.1128/mSystems.00045-21.2 FIG S1 Plot showing the calculated number of viral gene copies/liter of wastewater compared to the daily active COVID-19 caseload. Download FIG S1, PDF file, 0.4 MB. Copyright © 2021 Karthikeyan et al. 2021 Karthikeyan et al. https://creativecommons.org/licenses/by/4.0/ This content is distributed under the terms of the Creative Commons Attribution 4.0 International license.

The efficiency of high-throughput concentration method was compared to the two other commonly implemented concentration methods (electronegative membrane filtration and polyethylene glycol [PEG]-based) using ninefold serial dilutions of heat-inactivated SARS-CoV-2 viral particles spiked into 10-ml volumes of raw sewage which was previously verified to be from a location with no SARS-CoV-2 prevalence and verified by RT-qPCR (see Text S1 in the supplemental material). PMMoV (pepper mild mottle virus) concentration was used to normalize the obtained SARS-CoV-2 viral concentrations (to account for load changes, if any). The high-throughput protocol compared favorably to the conventionally used protocols demonstrating its potential implications for large-scale sample processing (>100 samples/day) (see Fig. S2A, Fig. S3, and Table S1 in the supplemental material). The average viral recovery efficiencies were 27% (standard deviation [SD], 8%), 15% (SD, 9%) and 13% (SD, 8%), respectively, for the high-throughput, PEG, and HA filtration protocol (Text S1). The improved recovery could also be attributed in part due to the specificity of the magnetic beads in recovering viral particles coupled with a magnetic-bead-based nucleic acid extraction protocol as well. The protocol was optimized on the KingFisher for optimal bead binding through homogenization (https://doi.org/10.17504/protocols.io.bptemnje). Furthermore, the automated protocol was hands-free and less prone to user discrepancies than the other two methods which were carried out manually.

10.1128/mSystems.00045-21.1 TEXT S1 Methods for wastewater viral concentration and detection. Download Text S1, DOCX file, 0.02 MB. Copyright © 2021 Karthikeyan et al. 2021 Karthikeyan et al. https://creativecommons.org/licenses/by/4.0/ This content is distributed under the terms of the Creative Commons Attribution 4.0 International license.

10.1128/mSystems.00045-21.3 FIG S2 (A) Viral concentration method comparison. RT-qPCR N1 Cq values for ninefold serial dilutions of heat-inactivated SARS-CoV-2 viral particles seeded into 10-ml volumes of raw sewage processed using the PEG concentration protocol (blue), magnetic-bead-based, Nanotrap protocol (purple), and electronegative membrane filtration method (gray). (B to D) Standard curves for N1, N2, and E gene, respectively, for sixfold serial dilutions of heat-inactivated SARS-CoV-2 viral particles spiked into 10 ml of raw sewage and concentrated using the high-throughput pipeline. Results for three replicates are shown. Download FIG S2, PDF file, 0.6 MB. Copyright © 2021 Karthikeyan et al. 2021 Karthikeyan et al. https://creativecommons.org/licenses/by/4.0/ This content is distributed under the terms of the Creative Commons Attribution 4.0 International license.

10.1128/mSystems.00045-21.4 FIG S3 Average N1 Cq values for 24 samples run with all three concentration methods. Download FIG S3, PDF file, 0.05 MB. Copyright © 2021 Karthikeyan et al. 2021 Karthikeyan et al. https://creativecommons.org/licenses/by/4.0/ This content is distributed under the terms of the Creative Commons Attribution 4.0 International license.

10.1128/mSystems.00045-21.6 TABLE S1 N1 Cq values for the 24 samples concentrated by the three methods. SD (%) shows the standard deviation of the two replicates per sample run. Download Table S1, DOCX file, 0.1 MB. Copyright © 2021 Karthikeyan et al. 2021 Karthikeyan et al. https://creativecommons.org/licenses/by/4.0/ This content is distributed under the terms of the Creative Commons Attribution 4.0 International license.

**FIG 1:**
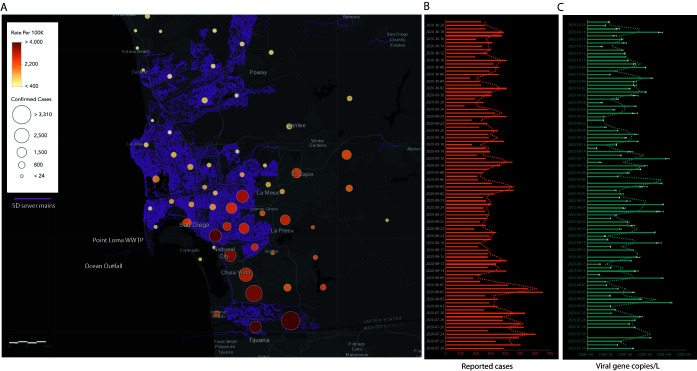
Tracking infection dynamics in San Diego County. (A) Map showing the San Diego sewer mains (depicted in purple) that feed into the influent stream at the primary wastewater treatment plant (WWTP) at Point Loma. Overlaid are the cumulative cases recorded from the different zip codes in the county during the course of the study. The caseload was counted by cases per zip code from areas draining into the WWTP. The sizes of the circles are proportional to the diagnostic cases reported from each zone, and the color gradient shows the number of cases per 100,000 residents. (B) Daily new cases reported by the county of San Diego. (C) SARS-CoV-2 viral gene copies detected per liter of raw sewage determined from N1 Cq values corrected for PMMoV (pepper mild mottle virus) concentration. All viral concentration estimates were derived from the processing of two sample replicates and two PCR replicates for each sample (error bars show the standard deviations [SD]).

**FIG 2:**
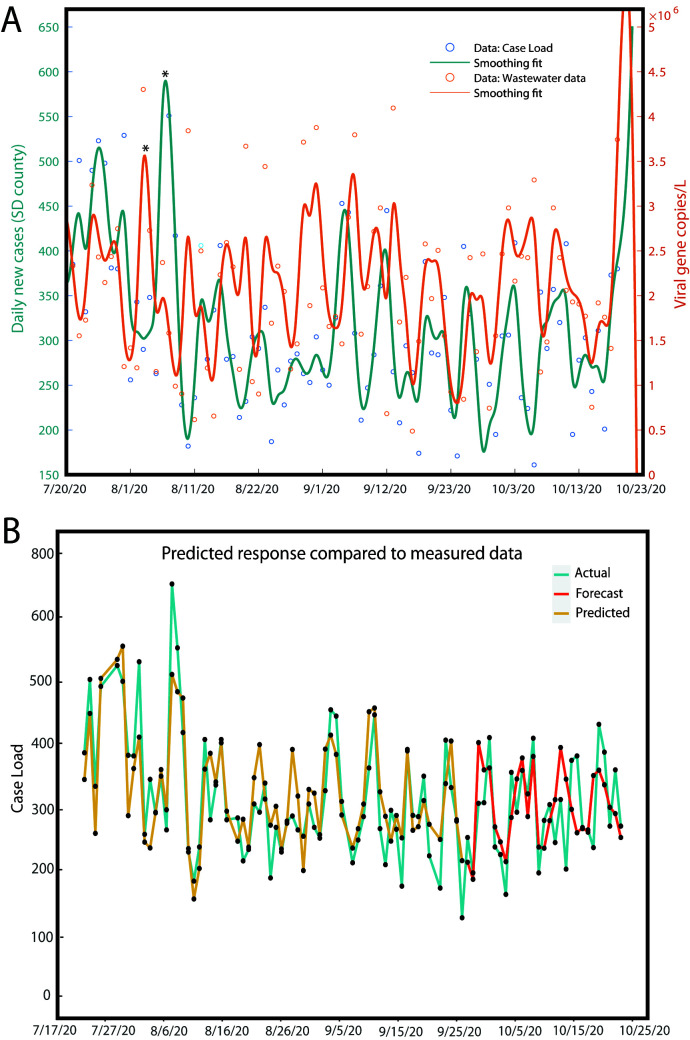
(A) Daily caseload and wastewater viral concentration data shown for a period of 13 weeks, where a spline smoothing is applied to each time series to demonstrate general trends. (B) Predictive model showing the predicted data (yellow) compared to the observed caseload (blue) and the 4-week forecast (red). Data collected from 07/07/2020 to 09/28/2020 were used as the training data set to predict the caseload for the following weeks (up to 10/25/2020). Data (wastewater plus county testing data) gathered from 09/29/2020 to 10/21/2020 were used for model validation. MATLAB Systems Identification toolbox was used to estimate the model order and parameters and calculate the forecasted values.

Twenty-four-hour flow weighted composites were collected each day for 3 months between 20 July 2020 and 21 October 2020 from the influent stream of Point Loma wastewater treatment plant. The plant processes over 175 MGD (million gallons per day) of raw sewage and is the primary treatment center for the greater San Diego area serving over 2.3 million residents. SARS-CoV-2 viral RNA was detected in all of the samples processed at an average concentration of 2,010,104 gene copies/liter in the influent. Over the course of the study, the clinically reported cases in the county increased by 29,375 (Fig. 1). Peaks in the wastewater data were frequently followed by peaks in the clinically confirmed cases at a later date. This suggests a correlation between wastewater and the number of new cases with the caveat of a time delay, where the wastewater data predicts future trends in the new number of cases. Although informative, this time-lagged correlation alone is not enough for robust predictions. This served as the main motivation to build a predictive model for forecasting the number of new cases per day in San Diego County. In order to model the number of new reported cases, which correlates not only to wastewater but also to other complex prevalence and spreading dynamics, we embed useful predictors as separate time series to take advantage of any side information that may improve our forecasting results. Here, the day of the week was tracked for each data point in a third time series to capture any weekly trends for a total of three time series with 88 data points each. We used a data-driven approach to train a prediction model that utilizes prior reported cases, wastewater data, and temporal correlations (embedded in the day of the week) in order to forecast the number of new positive cases in San Diego County. The multivariate autoregressive integrated moving average (ARIMA) model (7) was applied to build a prediction model for the number of new positive cases. The (predicted) number of new cases consist of lagged past values from all three series (number of new cases, wastewater data, day of the week), and each term can be thought of as the influence of that lagged time series on the number of new cases. A total of 65 data points corresponding to data collected from 7 July 2020 to 28 September 2020 were used for developing the predictive model and forecasting estimates. The remaining 24 data points, corresponding to data collected from 29 September 2020 to 25 October 2020, were reserved for validating our forecast estimates. Figure 2B shows the results of the model (prediction and forecast) compared to the observed data. The Pearson correlation coefficients (*r*) and root mean squared error (RMSE) between the observed data and the predicted model were *r* = 0.84, 0.79, 0.69, and 0.47 and RMSE = 57, 50, 59, and 70 for the trained model, 1, 2, and 3 week advance forecast values, respectively (Table S2). Our data-driven approach obtains forecasts successfully capturing general trends on the number of new cases (shown here up to 3 weeks in advance), ultimately reinforcing that wastewater analysis can be expository of previously undetected SARS-CoV-2 infections in the population which could provide useful insight for county officials for the purpose of early public health interventions. As with most environmental samples, there are biases and caveats associated with interpreting data from wastewater quantitatively, there are trade-offs while attempting to scale-up the process to enable the screening of hundreds of samples on a daily basis with a quick turnaround time (such as the requirement to purchase liquid-handling robots). However, our data show that we can still capture low prevalence cases enabling studies at high spatial resolution. Our study demonstrates that high-throughput wastewater-based surveillance can be successfully leveraged to enable creation of a rapid, large-scale early alert system for counties/districts and could be particularly useful in community surveillance in the more vulnerable populations.

10.1128/mSystems.00045-21.7 TABLE S2 Observed and predicted response values for the daily number of cases in San Diego County. Download Table S2, DOCX file, 0.02 MB. Copyright © 2021 Karthikeyan et al. 2021 Karthikeyan et al. https://creativecommons.org/licenses/by/4.0/ This content is distributed under the terms of the Creative Commons Attribution 4.0 International license.

10.1128/mSystems.00045-21.5 FIG S4 One-dimensional (1D) amplitude pictures of 2019nCOV ORF1a_ FAM. Download FIG S4, PDF file, 0.4 MB. Copyright © 2021 Karthikeyan et al. 2021 Karthikeyan et al. https://creativecommons.org/licenses/by/4.0/ This content is distributed under the terms of the Creative Commons Attribution 4.0 International license.

10.1128/mSystems.00045-21.8 TABLE S3 (A) Cycling conditions for RT-qPCR used for SARS-CoV-2 viral RNA detection using the multiplex Promega protocol. (B) Cycling conditions for one-step ddPCR for viral quantification of the ORF1ab gene in the heat-inactivated SARS-CoV-2 viral particles used in recovery experiments. (C) Dilution series of the heat-inactivated SARS-CoV-2 viral particles used in recovery experiments. Download Table S3, DOCX file, 0.2 MB. Copyright © 2021 Karthikeyan et al. 2021 Karthikeyan et al. https://creativecommons.org/licenses/by/4.0/ This content is distributed under the terms of the Creative Commons Attribution 4.0 International license.
