# Supplementary figures and images for: High-Throughput Wastewater SARS-CoV-2 Detection Enables Forecasting of Community Infection Dynamics in San Diego County
Source: mSystems. 2021 Mar 2;6(2):e00045-21. doi: 10.1128/mSystems.00045-21 (PMC8546963; doi:10.1128/mSystems.00045-21)

# SARS-CoV-2 concentration in sewage and hospital daily caseload

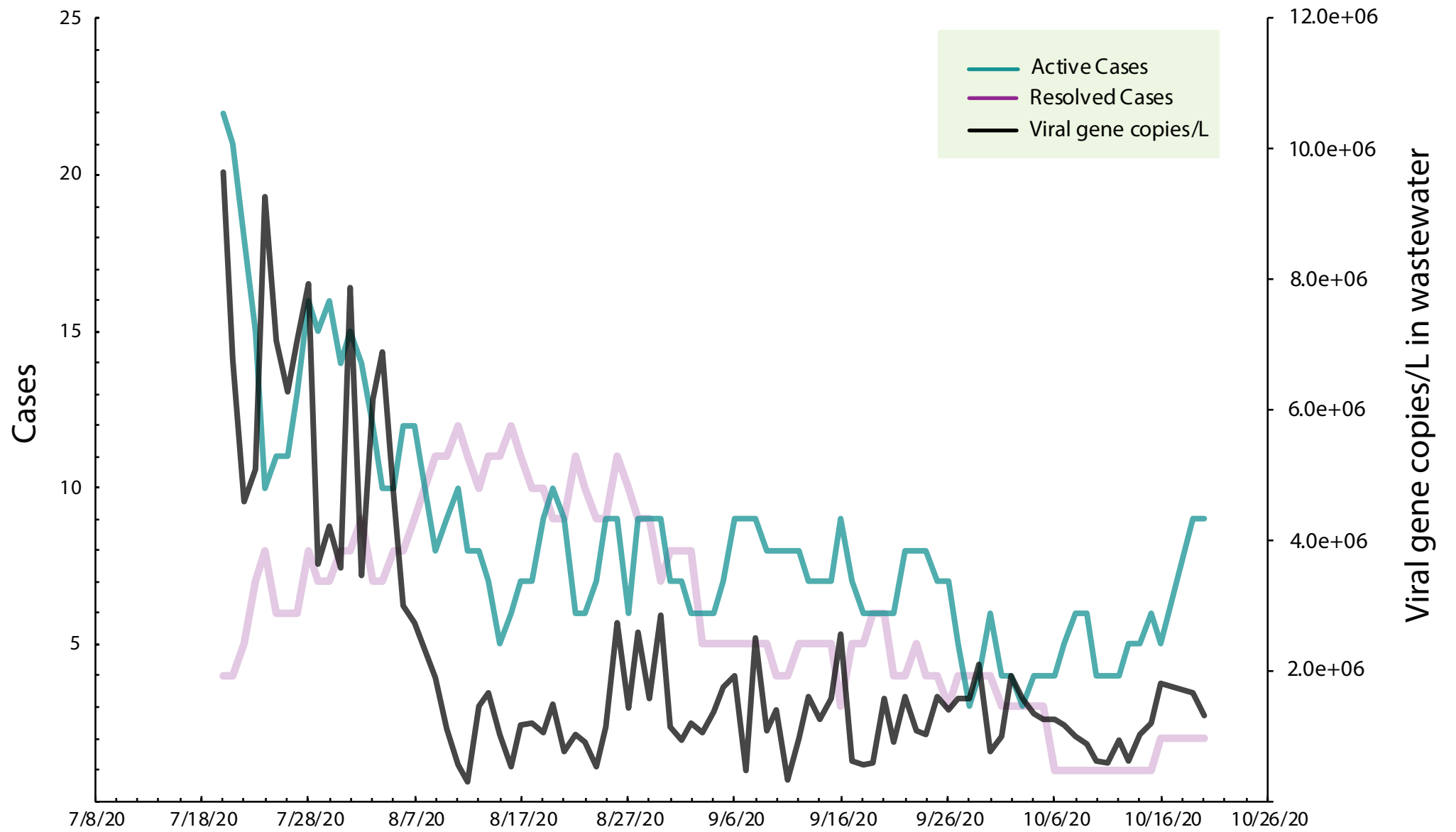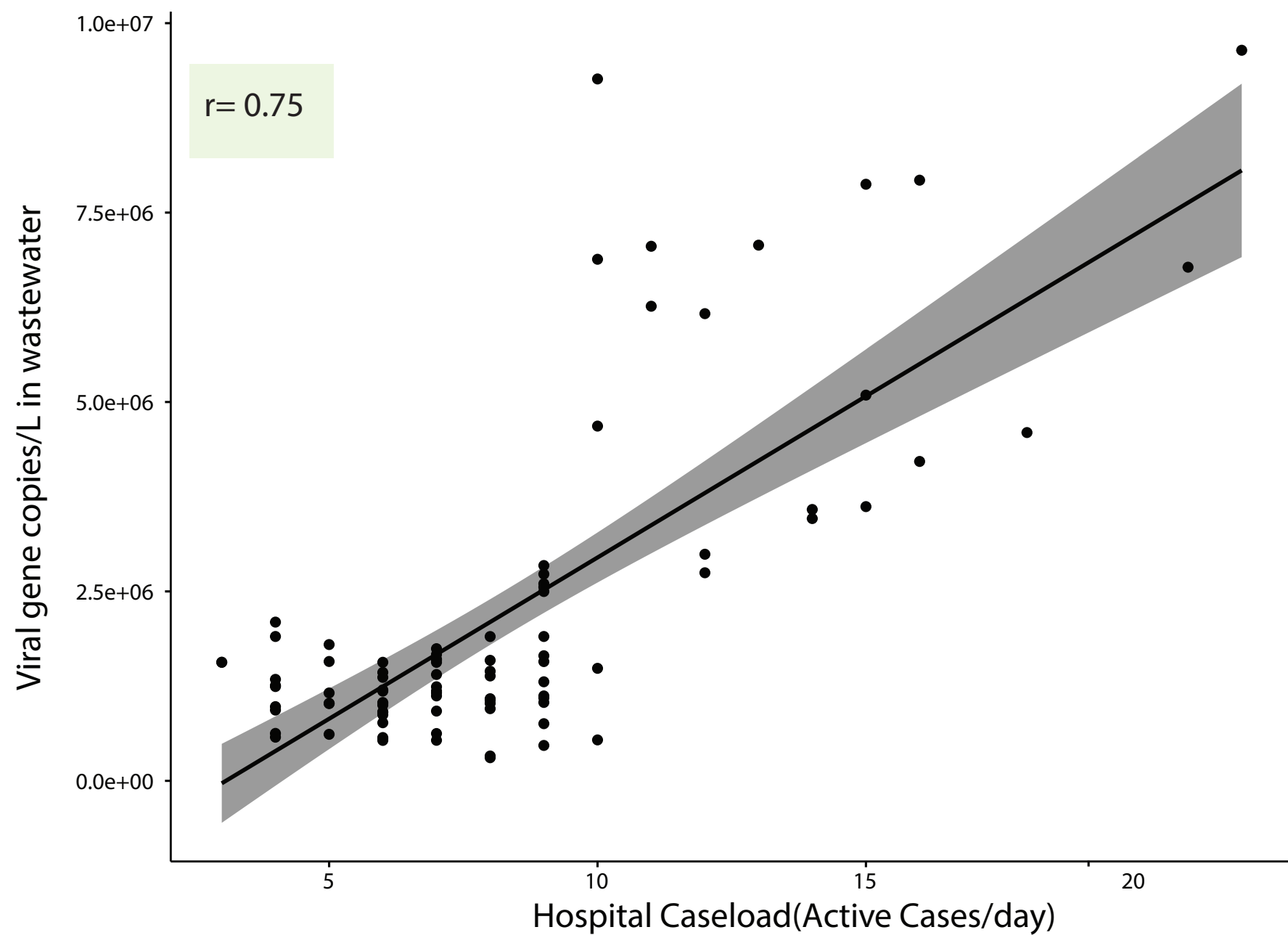

Supplement: FIG S1 [file msystems.00045-21-sf001.pdf]

A

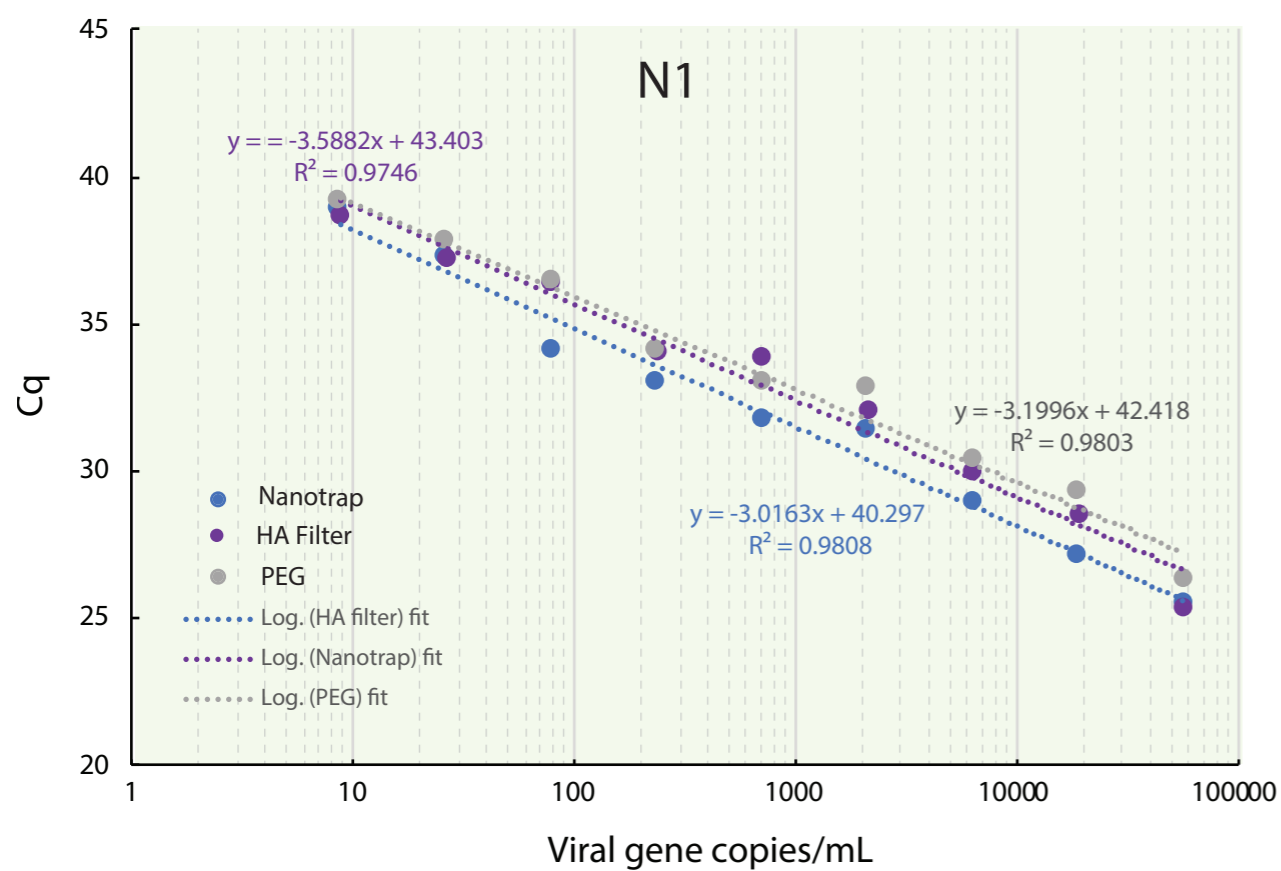

B

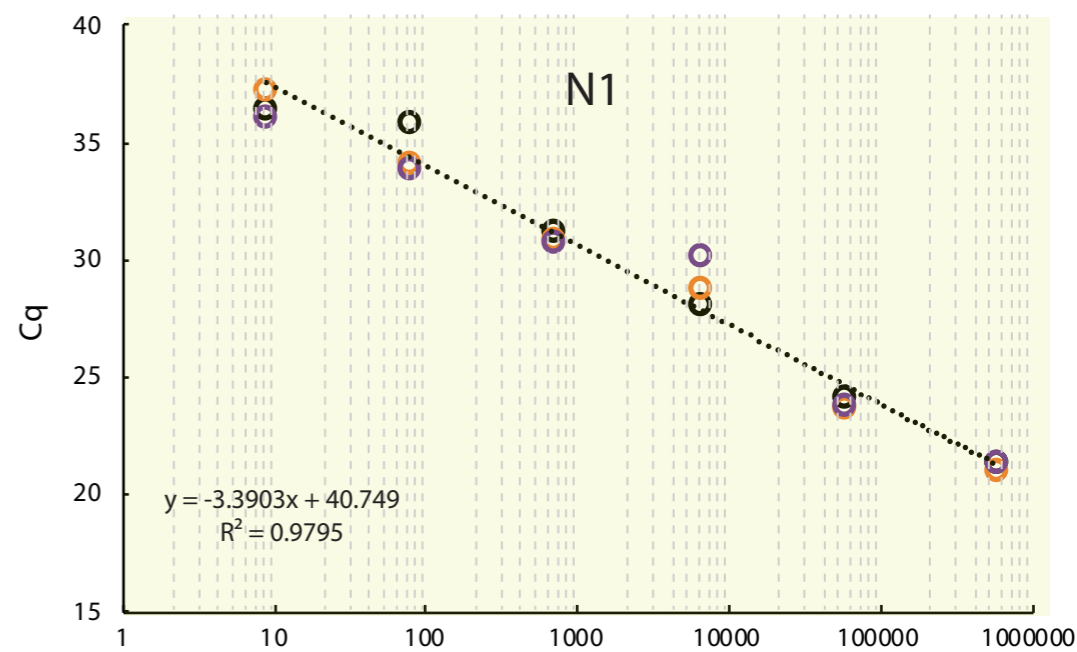

C

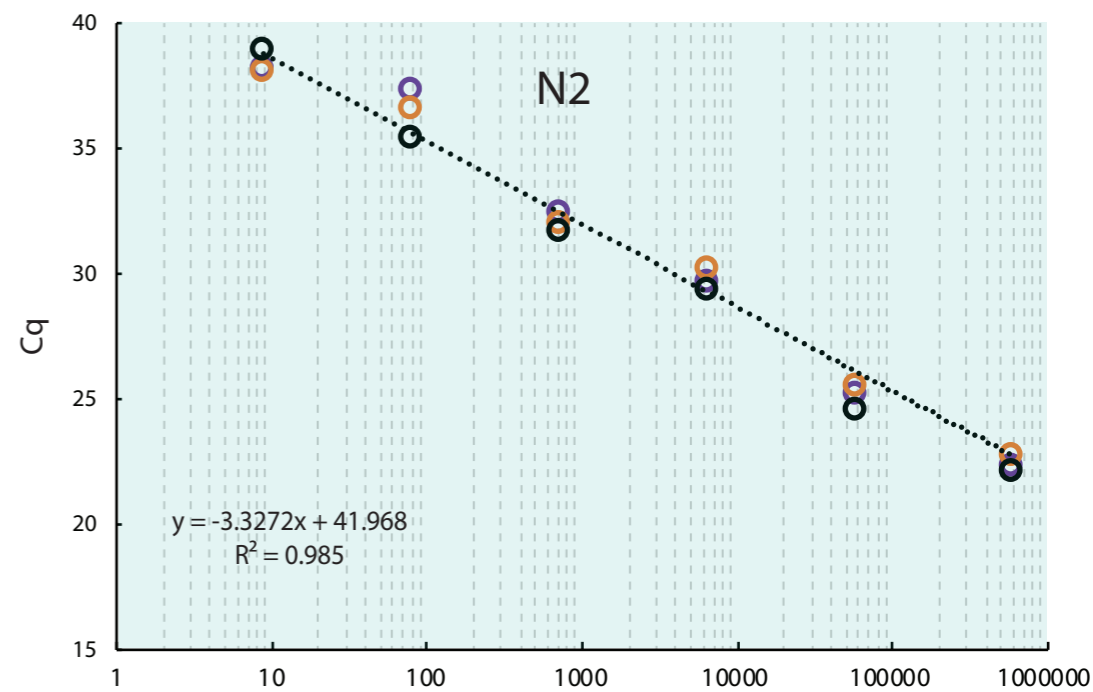

D

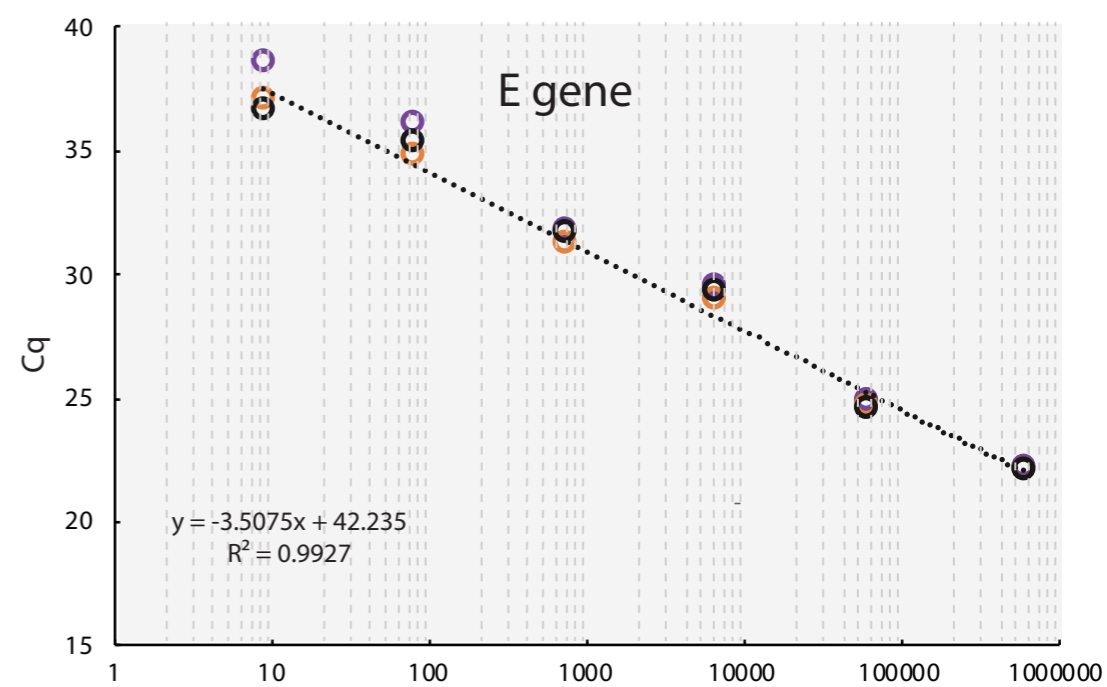

Supplement: FIG S2 [file msystems.00045-21-sf002.pdf]

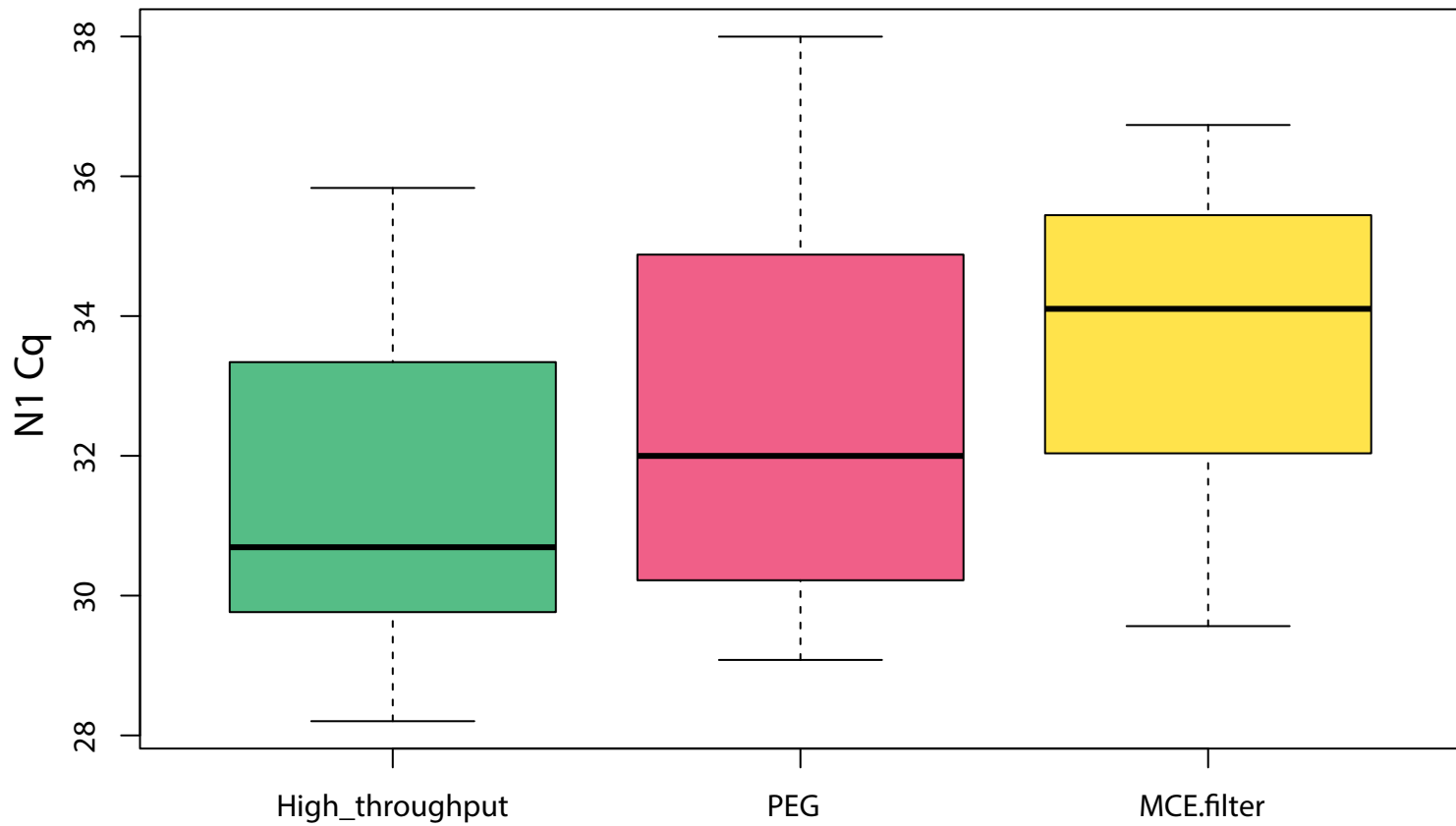

Supplement: FIG S3 [file msystems.00045-21-sf003.pdf]

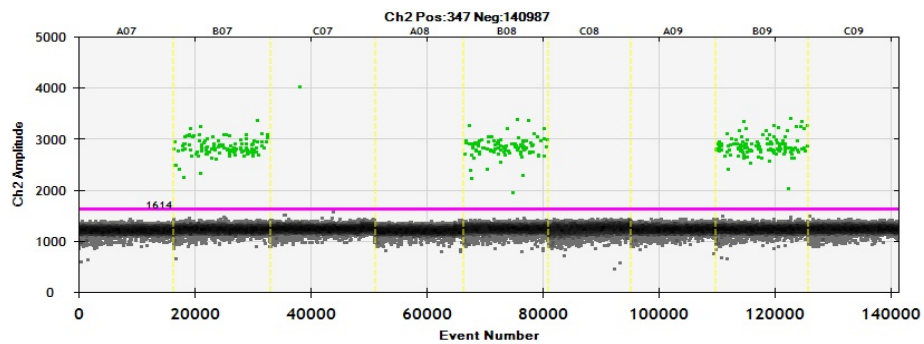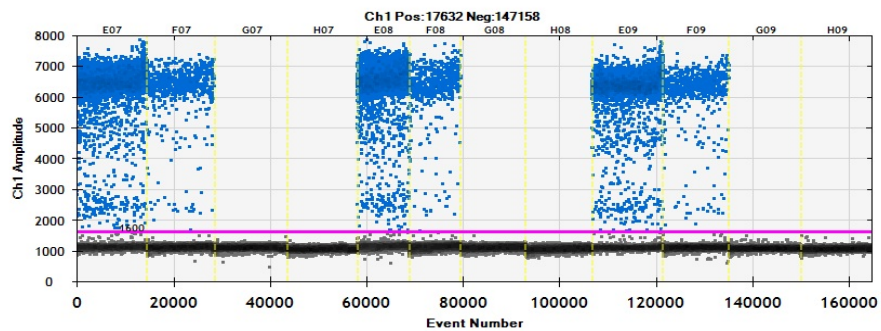

Supplement: FIG S4 [file msystems.00045-21-sf004.pdf]
