# Supplementary material for: High-Throughput Wastewater SARS-CoV-2 Detection Enables Forecasting of Community Infection Dynamics in San Diego County
Source: mSystems. 2021 Mar 2;6(2):e00045-21. doi: 10.1128/mSystems.00045-21 (PMC8546963; doi:10.1128/mSystems.00045-21)
Supplement: TABLE S2 [file msystems.00045-21-st002.docx]

|  | **Daily new cases (SD county)** | | | **Viral gene copies/L** | **Day of the week** |
| --- | --- | --- | --- | --- | --- |
| **Date** | Data | Estimated Model | Forecast values | Wastewater data | 1=Monday |
| 7/20/20 | 385 | 342.7569 |  | 2,332,026.54 | 1 |
| 7/21/20 | 501 | 447.1958 |  | 1,551,857.02 | 2 |
| 7/22/20 | 332 | 257.5234 |  | 1,727,150.62 | 3 |
| 7/23/20 | 490 | 502.6874 |  | 3,235,187.12 | 4 |
| 7/26/20 | 523 | 532.747 |  | 2,431,730.76 | 7 |
| 7/27/20 | 498 | 553.6315 |  | 2,146,578.31 | 1 |
| 7/28/20 | 381 | 285.5421 |  | 2,437,051.95 | 2 |
| 7/29/20 | 380 | 360.0118 |  | 2,747,828.41 | 3 |
| 7/30/20 | 529 | 409.7799 |  | 1,207,764.74 | 4 |
| 7/31/20 | 256 | 243.7113 |  | 1,417,154.42 | 5 |
| 8/1/20 | 343 | 234.4321 |  | 1,194,551.65 | 6 |
| 8/2/20 | 290 | 290.8771 |  | 4,303,251.63 | 7 |
| 8/3/20 | 348 | 358.3898 |  | 2,728,333.35 | 1 |
| 8/4/20 | 263 | 294.8408 |  | 1,152,773.68 | 2 |
| 8/5/20 | 652 | 508.8962 |  | 2,369,773.37 | 3 |
| 8/6/20 | 551 | 481.5993 |  | 1,582,051.11 | 4 |
| 8/7/20 | 417 | 471.6133 |  | 988,905.61 | 5 |
| 8/8/20 | 228 | 233.411 |  | 904,293.88 | 6 |
| 8/9/20 | 182 | 153.7541 |  | 3,842,016.87 | 7 |
| 8/10/20 | 236 | 201.9887 |  | 616,073.46 | 1 |
| 8/11/20 | 406 | 359.0887 |  | 2,500,428.42 | 2 |
| 8/12/20 | 279 | 384.1577 |  | 1,192,246.95 | 3 |
| 8/13/20 | 334 | 338.8829 |  | 655,394.59 | 4 |
| 8/14/20 | 406 | 400.727 |  | 2,233,170.30 | 5 |
| 8/15/20 | 279 | 292.5592 |  | 2,592,742.60 | 6 |
| 8/17/20 | 282 | 245.1322 |  | 2,320,963.19 | 1 |
| 8/18/20 | 214 | 279.8132 |  | 1,177,421.34 | 2 |
| 8/19/20 | 232 | 235.8228 |  | 3,669,143.04 | 3 |
| 8/20/20 | 304 | 345.5726 |  | 1,040,025.39 | 4 |
| 8/21/20 | 291 | 397.916 |  | 901,987.27 | 5 |
| 8/22/20 | 337 | 311.7353 |  | 3,442,221.79 | 6 |
| 8/23/20 | 187 | 270.4436 |  | 1,692,578.54 | 7 |
| 8/24/20 | 267 | 300.3693 |  | 2,330,169.78 | 1 |
| 8/25/20 | 228 | 232.7673 |  | 2,046,962.79 | 2 |
| 8/26/20 | 277 | 273.5418 |  | 1,180,550.83 | 3 |
| 8/27/20 | 285 | 390.2893 |  | 1,461,755.50 | 4 |
| 8/28/20 | 263 | 316.5342 |  | 3,714,923.59 | 5 |
| 8/29/20 | 253 | 198.707 |  | 1,888,830.98 | 6 |
| 8/30/20 | 304 | 327.0192 |  | 3,877,063.78 | 7 |
| 8/31/20 | 267 | 321.109 |  | 2,086,813.19 | 1 |
| 9/1/20 | 250 | 255.4555 |  | 1,655,486.25 | 2 |
| 9/2/20 | 325 | 390.839 |  | 1,767,155.04 | 3 |
| 9/3/20 | 453 | 412.9572 |  | 1,461,755.50 | 4 |
| 9/4/20 | 443 | 382.3747 |  | 2,875,872.26 | 5 |
| 9/5/20 | 308 | 286.561 |  | 3,796,334.60 | 6 |
| 9/7/20 | 211 | 234.3069 |  | 1,570,126.24 | 1 |
| 9/8/20 | 247 | 264.8406 |  | 2,102,392.59 | 2 |
| 9/9/20 | 284 | 303.8505 |  | 2,716,640.54 | 3 |
| 9/10/20 | 361 | 449.9258 |  | 2,980,097.51 | 4 |
| 9/11/20 | 445 | 455.8139 |  | 682,574.24 | 5 |
| 9/12/20 | 265 | 323.2479 |  | 4,095,336.32 | 6 |
| 9/13/20 | 208 | 284.5112 |  | 1,705,893.20 | 7 |
| 9/14/20 | 294 | 245.2321 |  | 2,204,297.45 | 1 |
| 9/15/20 | 264 | 285.8503 |  | 487,224.07 | 2 |
| 9/16/20 | 174 | 250.3876 |  | 1,490,048.76 | 3 |
| 9/17/20 | 388 | 390.2926 |  | 2,578,090.07 | 4 |
| 9/18/20 | 286 | 262.3267 |  | 1,966,959.29 | 5 |
| 9/19/20 | 284 | 268.0696 |  | 2,505,701.77 | 6 |
| 9/20/20 | 348 | 309.3863 |  | 1,552,473.50 | 7 |
| 9/21/20 | 222 | 271.8353 |  | 925,178.92 | 1 |
| 9/23/20 | 171 | 247.8889 |  | 816,590.99 | 3 |
| 9/24/20 | 405 | 336.211 |  | 843,455.66 | 4 |
| 9/25/20 | 330 | 403.6176 |  | 2,424,640.19 | 5 |
| 9/26/20 | 279 | 276.8502 |  | 1,373,821.46 | 6 |
| 9/27/20 | 124 | 214.3101 |  | 2,466,391.42 | 7 |
| 9/28/20 | 251 |  | 211.8419 | 743,916.77 | 1 |
| 9/29/20 | 195 |  | 184.8794 | 1,552,462.51 | 2 |
| 9/30/20 | 305 |  | 401.1089 | 2,466,391.42 | 3 |
| 10/1/20 | 306 |  | 357.901 | 2,980,097.51 | 4 |
| 10/2/20 | 409 |  | 361.1006 | 2,163,129.52 | 5 |
| 10/3/20 | 236 |  | 267.7269 | 2,441,459.52 | 6 |
| 10/4/20 | 224 |  | 244.0649 | 2,424,640.20 | 7 |
| 10/5/20 | 161 |  | 212.6933 | 3,292,463.35 | 1 |
| 10/6/20 | 354 |  | 282.607 | 1,147,833.24 | 2 |
| 10/7/20 | 291 |  | 343.6412 | 1,483,191.26 | 3 |
| 10/8/20 | 357 |  | 376.8217 | 2,980,097.51 | 4 |
| 10/9/20 | 320 |  | 379.2427 | 2,424,640.19 | 5 |
| 10/10/20 | 408 |  | 284.1227 | 2,059,575.59 | 6 |
| 10/11/20 | 195 |  | 235.8501 | 1,930,223.93 | 7 |
| 10/12/20 | 278 |  | 234.0864 | 1,907,001.33 | 1 |
| 10/13/20 | 303 |  | 277.6383 | 1,771,271.45 | 2 |
| 10/14/20 | 243 |  | 310.8178 | 753,837.19 | 3 |
| 10/15/20 | 311 |  | 393.3864 | 1,919,260.63 | 4 |
| 10/16/20 | 201 |  | 343.2658 | 1,759,581.55 | 5 |
| 10/17/20 | 373 |  | 295.4904 | 1,411,080.92 | 6 |
| 10/18/20 | 380 |  | 258.3931 | 3,742,657.88 | 7 |
| 10/19/20 | 265 |  | 266.7648 | 1,553,446.52 | 1 |
| 10/20/20 | 263 |  | 259.4196 | 909,420.24 | 2 |
| 10/21/20 | 235 |  | 348.9845 | 1,115,330.21 | 3 |
| 10/22/20 | 430 |  | 357.4664 |  | 4 |
| 10/23/20 | 386 |  | 334.3438 |  | 5 |
| 10/24/20 | 269 |  | 298.7001 |  | 6 |
| 10/25/20 | 358 |  | 288.7466 |  | 7 |
| 10/26/20 | 269 |  | 251.0131 |  | 1 |
