# Supplementary material for: High-Throughput Wastewater SARS-CoV-2 Detection Enables Forecasting of Community Infection Dynamics in San Diego County
Source: mSystems. 2021 Mar 2;6(2):e00045-21. doi: 10.1128/mSystems.00045-21 (PMC8546963; doi:10.1128/mSystems.00045-21)
Supplement: Supplementary file 4 [file msystems.00045-21-st001.docx]

	High-throughput	PEG	HA filter	SD1 (%)	SD2 (%)	SD3 (%)
Sample 1	30.78692	34.05881	32.41462	1.4	0.6	1.9
Sample 2	34.23556	36.65705	35.14722	0.8	2.3	1.2
Sample 3	29.47480	29.07973	32.54974	0.5	5.2	3.4
Sample 4	30.59764	33.74786	35.81759	0.9	2.2	0.7
Sample 5	32.19717	36.47482	34.45838	2.4	1.2	3.3
Sample 6	34.13084	35.20022	35.74061	0.9	6.2	0.5
Sample 7	30.32122	34.09477	31.65733	1.8	3.2	2.6
Sample 8	32.39510	35.65271	33.01016	0.6	5.4	3.9
Sample 9	34.21794	34.55780	34.76957	0.2	0.9	1.1
Sample 10	35.83308	37.99780	36.73358	3.1	1.4	0.3
Sample 11	34.22338	35.62932	34.54974	0.6	2.7	1.3
Sample 12	29.15554	30.27448	30.61848	0.4	3.3	4.2
Sample 13	29.14301	29.38306	30.72101	0.3	0.9	2.8
Sample 14	28.20355	30.16491	29.56369	0.6	3.6	0.5
Sample 15	29.54997	31.00687	32.62336	1.2	2.9	4.4
Sample 16	34.05881	32.41462	36.06037	2.4	0.6	0.5
Sample 17	29.96500	29.17756	35.91373	2.1	1.8	5.6
Sample 18	31.58356	30.59764	33.74786	0.6	1.7	3.2
Sample 19	30.32122	34.09477	31.65733	0.5	0,4	6.6
Sample 20	29.96500	29.17756	35.91373	0.8	1.6	0.7
Sample 21	29.56369	29.54997	30.78692	1.1	0.6	3.5
Sample 22	32.62336	31.00687	34.85595	3.5	6.3	1.9
Sample 23	31.58356	30.59764	33.74786	3.6	4.8	3.2
Sample 24	30.12156	31.58356	34.96056	2.7	1.4	0.8
